# Keynote 2: Do we need a concept of sustainable HEPA?

**DOI:** 10.1093/eurpub/ckad133.002

**Published:** 2023-09-11

**Authors:** Karim Abu-Omar

**Affiliations:** Department of Sports Science and Sport at Friedrich-Alexander-Universität Erlangen-Nürnberg (FAU), Germany

## Abstract

Climate change and the need to reduce global carbon emissions drastically in upcoming years pose a challenge to physical activity (PA) promotion. Not only is PA more difficult in heat and people need to adapt, but also many physical activities come with a comparable high carbon footprint. While the field of PA promotion has so far utilized the health-enhancing physical activity (HEPA) concept to define and promote PA, this concept might need to be amended to account for the environmental impacts of PA. The aim of the presentation is to critically analyze the interconnections between PA and planetary health and suggest potential steps towards a concept of sustainable HEPA.

